# The Zambian Wildlife Ranching Industry: Scale, Associated Benefits, and Limitations Affecting Its Development

**DOI:** 10.1371/journal.pone.0081761

**Published:** 2013-12-18

**Authors:** Peter A. Lindsey, Jonathan Barnes, Vincent Nyirenda, Belinda Pumfrett, Craig J. Tambling, W. Andrew Taylor, Michael t’Sas Rolfes

**Affiliations:** 1 Lion Program, Panthera, New York, New York, United States of America; 2 Mammal Research Institute, Department of Zoology and Entomology, University of Pretoria, Pretoria, Gauteng, South Africa; 3 Design & Development Services, Windhoek, Khomas Region, Namibia; 4 Zambia Wildlife Authority, Chilanga, Lusaka Province, Zambia; 5 Kafue Trust, Lusaka, Lusaka Province, Zambia; 6 Centre for African Conservation Ecology, Department of Zoology, Nelson Mandela Metropolitan University, Port Elizabeth, Eastern Cape, South Africa; 7 Centre for Veterinary Wildlife Studies, Faculty of Veterinary Sciences, University of Pretoria, Pretoria, Gauteng, South Africa; 8 Independent consultant, Cape Town, Western Cape, South Africa; University of Kent, United Kingdom

## Abstract

The number and area of wildlife ranches in Zambia increased from 30 and 1,420 km^2^ in 1997 to 177 and ∼6,000 km^2^ by 2012. Wild ungulate populations on wildlife ranches increased from 21,000 individuals in 1997 to ∼91,000 in 2012, while those in state protected areas declined steeply. Wildlife ranching and crocodile farming have a turnover of ∼USD15.7 million per annum, compared to USD16 million from the public game management areas which encompass an area 29 times larger. The wildlife ranching industry employs 1,200 people (excluding jobs created in support industries), with a further ∼1,000 individuals employed through crocodile farming. Wildlife ranches generate significant quantities of meat (295,000 kg/annum), of which 30,000 kg of meat accrues to local communities and 36,000 kg to staff. Projected economic returns from wildlife ranching ventures are high, with an estimated 20-year economic rate of return of 28%, indicating a strong case for government support for the sector. There is enormous scope for wildlife ranching in Zambia due to the availability of land, high diversity of wildlife and low potential for commercial livestock production. However, the Zambian wildlife ranching industry is small and following completion of field work for this study, there was evidence of a significant proportion of ranchers dropping out. The industry is performing poorly, due to *inter alia*: rampant commercial bushmeat poaching; failure of government to allocate outright ownership of wildlife to landowners; bureaucratic hurdles; perceived historical lack of support from the Zambia Wildlife Authority and government; a lack of a clear policy on wildlife ranching; and a ban on hunting on unfenced lands including game ranches. For the wildlife ranching industry to develop, these limitations need to be addressed decisively. These findings are likely to apply to other savanna countries with large areas of marginal land potentially suited to wildlife ranching.

## Introduction

Wildlife management in southern Africa has evolved through several phases [1] With European settlement, wildlife populations were decimated by unregulated hunting, habitat fragmentation and diseases [2]. A protectionist phase followed whereby colonial governments centralized control over wildlife and limited commercial use, making wildlife a burden for landowners [3]. Wildlife waned further due to poaching, persecution by landowners to reduce competition with livestock, state-sponsored hunting to remove tsetse fly *Glossina* spp. hosts and construction of veterinary fences [4],[5],[6]. Negative wildlife population trends improved following legislative changes made during the 1960–70s that granted landowners the right to utilize wildlife on their land [6]. These changes coincided with rising demand for tourism and safari hunting, recurrent droughts, declining range productivity due to overstocking with livestock, and declining state subsidies for livestock production [7],[8],[9]. These factors contributed to a massive shift from livestock farming to wildlife ranching in parts of the region. In Zimbabwe, for example, prior to the land seizures in 2000, 1,000 wildlife ranches had developed, covering 27,000 km^2^ (Bond et al., 2004). In South Africa, there are >9,000 wildlife ranches, covering 205,000 km^2^ and an additional 15,000 mixed livestock and wildlife ranches [10] and in Namibia, wildlife ranches encompass 288,000 km^2^ [11].

Wildlife is a productive form of land use on marginal lands where alternatives such as agriculture are not viable [12]. Wildlife ranching can be developed in conjunction with, or in place of livestock farming and confers a wide range of economic, social and ecological benefits [13]. Wildlife ranching can contribute significantly to food security though the generation of significant quantities of protein, through employment, and through generation of foreign currency [11]. Zambia has massive potential for developing wildlife ranching, on leasehold land, on communal land and through exploiting the full potential of the 167,000 km^2^ of game management areas (GMAs) and vast areas of other potentially suitable land [14]. The country has an extremely diverse array of wildlife and much of Zambia is poorly suited to agriculture and livestock due to the presence of leached soils, regular droughts and widespread tsetse fly.

Wildlife ranching has potential to contribute significantly to wildlife conservation efforts in Zambia. Zambia has set aside a vast area of land for wildlife conservation: the protected area network encompasses 167,000 km^2^ of game management areas and 64,000 km^2^ of national parks. However, the Zambia Wildlife Authority does not have the resources to protect those areas effectively and most protected areas are performing poorly due *inter alia* to bushmeat poaching and encroachment of protected areas [15]–[17]. Accordingly, wildlife ranching is recognized in principle by the government in Zambia as offering scope to provide an insurance policy for the national protected areas network, and as a key option for the utilization of unproductive lands [18]. However, in reality the Zambian wildlife ranching industry is greatly hampered by an inadequate and inappropriate legislative and policy framework, inadequate legal protection from poaching, and government bureaucracy, and is small and under-developed as a result [14].

This paper sets out to assess the current scale and associated benefits of the game ranching industry in Zambia and to identify constraints currently limiting development of the industry. The findings of this paper are likely to apply to varying degrees to the many other savanna African countries with vast marginal lands potentially suited to wildlife ranching and with variable performance of traditional protected area networks.

## Results

We present three categories of information, first a review of the legislative and administrative basis for wildlife ranching; second a descriptive overview of the industry and perceptions regarding benefits and limitations; and finally the results of a model which assess the financial and economic productivity of a wildlife ranching enterprise.

### Legislative and Administrative Basis for Wildlife Ranching in Zambia

Private land in Zambia is leased to individuals/companies for 99-year periods [14]. Wildlife ranching is governed by the Zambia Wildlife Act No. 12 of 1998 and the Policy for Wildlife and National Parks [19]. However, both documents barely mention wildlife ranching and simply outline conditions under which wildlife ranches may be established. The Wildlife Act identifies ZAWA as the authority responsible for regulating wildlife ranching and stipulates that wildlife in Zambia is owned by the president on behalf of the nation. Provision is made for allocation of certificates of ownership of wildlife and permits to keep wild animals in captivity to landowners whose wild animals have been counted and property is encompassed by fencing. To obtain such certificates, ranchers are required to pay for wildlife on their land at gazetted prices, and apply for ‘wildlife ranch status’ via an application (which must include an ecological and business assessment by an external consultant) to a select committee [19]. When wildlife ranch status is bestowed upon a landowner s/he does not legally require approved quotas from ZAWA in order to utilize wildlife. To sell meat or live wildlife, veterinarians inspect animals/meat for diseases or parasites before issuing movement permits for movement between districts within Zambia or for exports to other countries. Wildlife ranchers are required to submit annual returns to ZAWA, which include information on wildlife numbers and numbers utilized. Such returns are the basis for reissuance of certificates of ownership of wildlife. Owners of unfenced wildlife ranches cannot obtain certificates of ownership for wildlife, and are required to apply to ZAWA for hunting quotas and to pay license fees for animals utilized. However, they are not required to obtain permits to keep wild animals in captivity.

According to the Wildlife Act, private individuals can apply to become Honorary Wildlife Police Officers. When in the presence of ZAWA scouts, Wildlife Police Officers are empowered to carry firearms, arrest people suspected of poaching on their land without a warrant, and confiscate weapons. The process may take as long as 3-months and costs ∼USD530 per scout. Individuals without Wildlife Police Officer status may not use firearms in the context of anti-poaching.

Wildlife ranchers wishing to practise ecotourism are required to pay the Zambia Tourism Board tourism operating licenses of USD400/year, business and liquor licenses of USD700/year and guides’ licenses of USD45–450/year (depending on the immigration status of the applicant). Those wishing to sell trophy hunts must pay USD2,500/year for a safari hunting operators’ license and USD800/year for a professional hunter’s license.

### Industry Indicators

**Figure 1:**
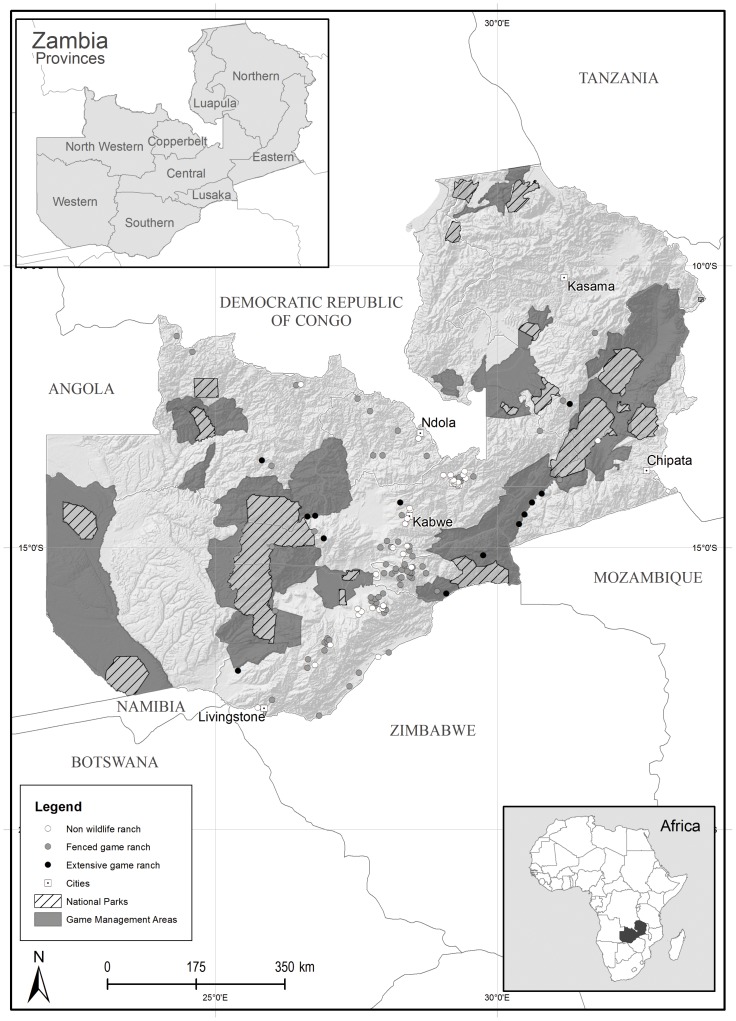
Location of properties of game ranchers and non-game ranchers surveyed.

The number of wildlife ranches registered with ZAWA has increased in recent years from 30 comprising 1,420 km^2^ in 1997 to 177 in 2012 comprising 5,981 km^2^ suggesting that the industry has expanded considerably (Figure 1). Approximately 3,155 km^2^ is comprised of extensive unfenced ranches. The 177 ranches included 115 ranches registered with ZAWA, 44 awaiting wildlife-ranch status, 11 other ranches not listed in the ZAWA database but practising wildlife-based land uses, and seven others in our survey about to apply for wildlife ranch status. It is important to note that 42.6% of wildlife ‘ranches’ were ornamental plots <2 km^2^ in size.

However, almost half of wildlife ranchers felt that the wildlife ranching industry was currently static or shrinking in size, due: the threat from poaching; inappropriate legislation and lack of government support; and failure to allocate full ownership of wildlife to ranchers (Table 1). Those disadvantages reduce the attractiveness of wildlife ranching. In 2013, after completion of the survey, there was evidence of a sharp contraction of the industry: by mid-2013, 54 ranchers had not renewed their wildlife ranch status, and despite ZAWA approving most pending wildlife ranch applications in early 2013, only two such ranchers actually followed-up to obtain permits. In addition, among non-wildlife ranchers, many would consider engaging in the industry if government addressed the limiting factors, and especially the poaching threat (Table 1). Six wildlife ranchers were in the process of removing wildlife due to poaching and perceived lack of support from the government.

**Table 1:** Perceived positives and negatives associated with game ranching as a land use, and perceptions on what would have to change for non-game ranchers to engage in wildlife-based land uses.

		% of ranchers	
Ranchers believing wildlife ranching to be increasing in prevalence		48.6%	
Ranchers believing wildlife ranching industry to be static in size		27.2%	
Ranchers believing wildlife ranching to be declining in prevalence		21.9%	
Ranchers who do not know what trends in the industry are		2.3%	
	**Game ranchers**	**Other ranchers**	**Stakeholders**
Perceived advantages/positive aspects of game ranching as a land use			
Conservation contribution	44.8%	46.8%	41.2%
Enables use of marginal land	22.9%	21.3%	29.4%
Food security/meat/forex/employment	17.7%	8.5%	35.3%
Profitable	32.3%	8.5%	11.8%
Perceived disadvantages and negative aspects of game ranching as a land use			
Poaching	86.0%	83.0%	58.8%
Red tape/lack of government support	71.0%	70.2%	52.9%
High capital start up costs	12.9%	48.9%	23.5%
Large land requirements/hard to get land	15.1%	23.4%	41.2%
Lack of clear ownership of wildlife	15.6%	17.0%	33.3%
Game ranching is not very profitable	9.5%	23.4%	11.1%
It is difficult to get bank loans for game ranching	6.5%	12.8%	5.9%
What would need to change for you to start game ranching?			
Poaching has to be better controlled		42.1%	
Would need financial support or to be in a stronger financial position		39.4%	
Less red tape/improved government support		31.5%	
Would need more land/government needs to make more land availablefor game ranching		26.3%	
Not interested		23.4%	

The mean size of extensive wildlife ranches in Zambia was 220±44 km^2^ (range 40–600 km^2^) while that of fenced wildlife ranches was 18.7±11.0 km^2^ (range 0.01–310 km^2^). All extensive wildlife ranches practised only wildlife-based land uses (though 33.3% were not financially active). Income generation on extensive ranches is limited to trophy hunting, ecotourism and unguided hunts, as live wildlife sales and shooting for meat production is not permitted (Table 2). Of fenced wildlife ranches, 54.0% utilized wildlife commercially, most commonly via trophy hunting, sale of venison and live wildlife, ecotourism and the sale of unguided hunting opportunities (Table 2). On wildlife ranches with mixed land use, wildlife yielded 23.3±23.5% of income. Infrastructure development for wildlife land uses was limited on most wildlife ranches and absent on many. For example, only 36.9% of wildlife ranches have developed accommodation for visiting hunters or tourists.

**Table 2:** Land use and income on extensive and fenced game ranches in Zambia.

	% of ranches generatingincome from land use	Mean % of individual ranchers’income (% wildlife income)	Mean % of total income (allranchers’ income combined)
Extensive game ranches* (n = 14)			
Livestock	0%	0%	0%
Agriculture	0%	0%	0%
Any form of commercial wildlife use	90.0%	100%	100%
% of wildlife income:			
Trophy hunting	77.8%	81.9%	88.7%
Ecotourism	55.6%	17.8%	10.9%
Sale of unguided hunts	22.2%	0.4%	0.4%
Live game sales	0%	0%	0%
Sale of game meat	0%	0%	0%
Fenced game ranches (n = 83)			
Livestock	47.6%	30.0%	52.5%
Agriculture	36.5%	31.9%	38.3%
Any form of commercial wildlife use	54.0%	38.1%	9.2%
% of wildlife income			
Trophy hunting	60.6%	31.2%	28.4%
Sale of game meat	54.6%	18.6%	10.2%
Live game sales	40.6%	18.6%	6.3%
Ecotourism	27.2%	20.1%	50.0%
Unguided hunts	25.7%	7.6%	5.0%

• Excluding ranches awaiting title.

On the extensive ranches that were financially active, mean gross earnings from wildlife were U$878±226/km^2^/year. On fenced wildlife ranches, gross earnings from wildlife (USD15,879±879/km^2^/year) were similar earnings from livestock (USD11,637±2,352/km^2^) (*F Ratio* = 0.009, d.f. = 1, *p = *0.923), though due to different cost structures net earnings may differ. Median earnings from wildlife per kg of wildlife biomass (USD0.61/kg) on fenced ranches were similar to those from livestock (USD0.57/kg) (Kruskal-Wallis Z = 0.344, *p = *0.730). Gross earnings from wildlife on wildlife ranches were higher among ranchers who practised multiple forms of wildlife use: ranches generating USD1–1,999/km^2^ employed 1.94±0.32 forms of wildlife use and those generating >US2,000/km^2^ 2.17±0.34 forms of wildlife use (*F Ratio* 31.5, d.f. = 2, p<0.001).

Estimated gross turnover of wildlife-based land uses on wildlife ranches in our sample was USD1.78 million/year for extensive ranches and USD9.1 million/year for fenced ranches. Extrapolating to include all ranches registered with ZAWA at the end of 2012, the mean value of the industry was USD1.82 million on extensive and USD9.3 million on fenced ranches. The majority of income on extensive ranches was derived from trophy hunting, whereas on fenced ranches ecotourism was the most important form of wildlife-based land use (Table 2). Wildlife capture teams generated USD1.1 million/year and crocodile farms USD3–4 million/year from the export of ∼43,000 skins (most recent estimate, anonymous crocodile farmer, pers. comm.).

When asked whether they consider wildlife or livestock ranching to be more profitable, 67.3% of ranchers answered livestock, due to the well-established market for livestock (45.0%); because wildlife is harder to harvest (28.3%); due to high beef prices (26.7%) and because penalties for livestock rustling are harsher than for wildlife poaching (11.7%). Seventeen percent (17.2%) of ranchers felt that wildlife was more profitable, due to the high profitability of trophy hunting (50.0%); because the costs of managing wildlife are lower (40.0%); and because wildlife breeds faster than cattle (30.0%).

Approximately 295,000 kg of venison is produced annually on wildlife ranches, 37.2% from trophy hunting (Table 3). The majority of venison is sold to butcheries or individual buyers (48.8%, ∼144,000 kg), given/sold to workers (∼61,000 kg), used for guests/family (∼36,000 kg) or given/sold to communities (∼36,000 kg). A maximum of 1.98 million kg of venison could be produced from wildlife ranches if wildlife was harvested at a rate equivalent to the maximum intrinsic rate of increase of each species. Ranchers sold venison for a mean price of USD5.0±0.31/kg, though meat dealers pay lower prices (USD3.55/kg) for carcasses if the animal was shot in the body. Both meat dealers surveyed indicated that demand for venison is considerably higher than the supply. Wildlife ranching has been in an expansion phase and so many ranchers may have been leaving wildlife populations to increase, rather than harvesting them maximally.

**Table 3:** The off-take of wildlife as trophies and for meat on Zambian game ranches and associated meat production.

	Hunted as trophies	Meat produced from trophy hunting	Hunted for meat*	Meat produced from meathunting	Totalhunted	Total meat produced	Population on game ranches	% harvested (animals hunted plus captured live)	Max potentialoff-take	Maximum potential meat production
Buffalo	34	11,900	2	586	36	12,486	2,107	1.71%	16.6%	13,003
Bushbuck	106	3,498	244	6,344	350	9,842	6,015	5.82%	44.1%	70,008
Bushpig	61	2,340	142	4,729	203	7,069	5,589	3.63%	35.6%	65,987
Duiker Blue	0	0	0	0	0	0	5,572	0.00%	84.0%	0
Duiker Com	62	579	42	364	104	943	936	11.09%	62.5%	6,107
Eland	42	13,944	101	28,583	143	42,527	1,558	9.17%	18.4%	81,108
Elephant	2	3,300	0	0	2	3,300	1,710	0.12%	10.3%	458,101
Giraffe	0	0	1	586	1	586	321	0.31%	13.8%	26,015
Grysbok	15	30	0	0	15	30	2,130	0.70%	91.1%	9,894
Hartebeest	60	5,008	24	1,783	84	6,791	2,051	4.11%	26.4%	40,036
Hippo	8	4,080	1	784	9	4,864	1,530	0.59%	12.5%	149,704
Impala	186	5,766	2,075	63,080	2,261	68,846	27,998	8.08%	39.8%	335,001
Klipspringer	0	0	0	0	0	0	560	0.00%	64.8%	2,178
Kudu	76	12,464	226	24,422	302	36,885	6,287	4.80%	25.6%	173,544
Lechwe	56	3,441	98	5,341	154	8,782	1,513	10.18%	34.3%	28,287
Nyala	2	122	0	0	2	122	95	2.10%	30.0%	1,340
Oribi	21	164	0	0	21	164	730	2.81%	64.6%	3,626
Ostrich	0	0	5	620	5	620	378	1.32%	?	?
Puku	104	4,363	368	13,653	472	18,016	4,904	9.62%	34.5%	62,686
Reedbuck	51	1,869	125	3,988	176	5,857	2735	6.42%	39.8%	34,682
Roan	31	4,619	0	0	31	4,619	1,647	1.88%	21.5%	48,902
Sable	84	10,329	6	726	90	11,055	3,682	2.45%	22.9%	102,052
Sitatunga	17	1,087	0	0	17	1,087	328	5.27%	38.9%	4,099
Steenbok	0	0	0	0	0	0	117	0.00%	63.0%	450
Tsessebe	8	639	5	323	13	962	410	3.24%	29.7%	7,871
Warthog	82	3,300	228	8,618	310	11,918	4,831	6.42%	38.1%	69,576
Waterbuck	67	9,514	87	10,467	153	19,981	2,987	5.12%	24.1%	87,236
Wildebeest	7	966	27	3,194	34	4,160	630	5.40%	26.5%	19,722
Zebra	38	6,308	38	6,688	76	12,996	2,060	3.69%	22.3%	80,757
	1,220	109,630	3,845	184,879	5,064	294,508	91,411			1,981,972

* Including animals shot on unguided hunts, and those culled by ranch management to produce meat.

Approximately 6,000 wild animals of 21 species were sold live in Zambia during 2003–2012. The first public wildlife auction was held in 2011. The second one, held in 2012 was considered to be a failure due to low levels of interest among buyers (reflecting the negative sentiments towards prospects of the industry among ranchers).

Established extensive wildlife ranchers employed a mean of 30.0±5.7 workers (0.17±0.04/km^2^), whereas fenced ranchers employed a mean of 10.1±1.4 employees (11.4±8.8/km^2^ or 1.1±0.45/km^2^ [excluding ranches smaller than 2 km^2^ and excluding ranches where wildlife is not used commercially) for wildlife-based land uses. The number of employees per km^2^ (excluding ranches <2 km^2^) was higher on ranches that practised ecotourism than those that did not (*F Ratio* = 7.64, d.f. = 1, *p = *0.006). Wildlife ranchers employed marginally more workers per unit area than livestock farmers (1.1±0.33/km^2^ c.f. 0.95±0.25/km^2^, d.f. = 1, F Ratio = 0.125, p = 0.725).

Wildlife ranchers in our sample employed 1,145 workers specifically for wildlife-based land uses. Extrapolating to include all wildlife ranches registered with ZAWA in 2012, at least 1,197 people are employed on wildlife ranches for wildlife-based land uses. The two crocodile farmers surveyed employed 197 individuals and estimated that the industry as a whole employs 1,000 people. The two wildlife capture companies and one taxidermist employ 36 permanent and 15 seasonal staff.

Thirteen percent (12.8%) of ranchers surveyed were indigenous Zambians, 4.1% were of Asian and 83.1% of European descent. With some exceptions, indigenous Zambians were owners of small-holdings (median ranch size 0.03 km^2^, mean 10.2± km^2^). Indigenous Zambians (who comprise ∼99.5% of the population) are thus under-represented.

**Figure 2:**
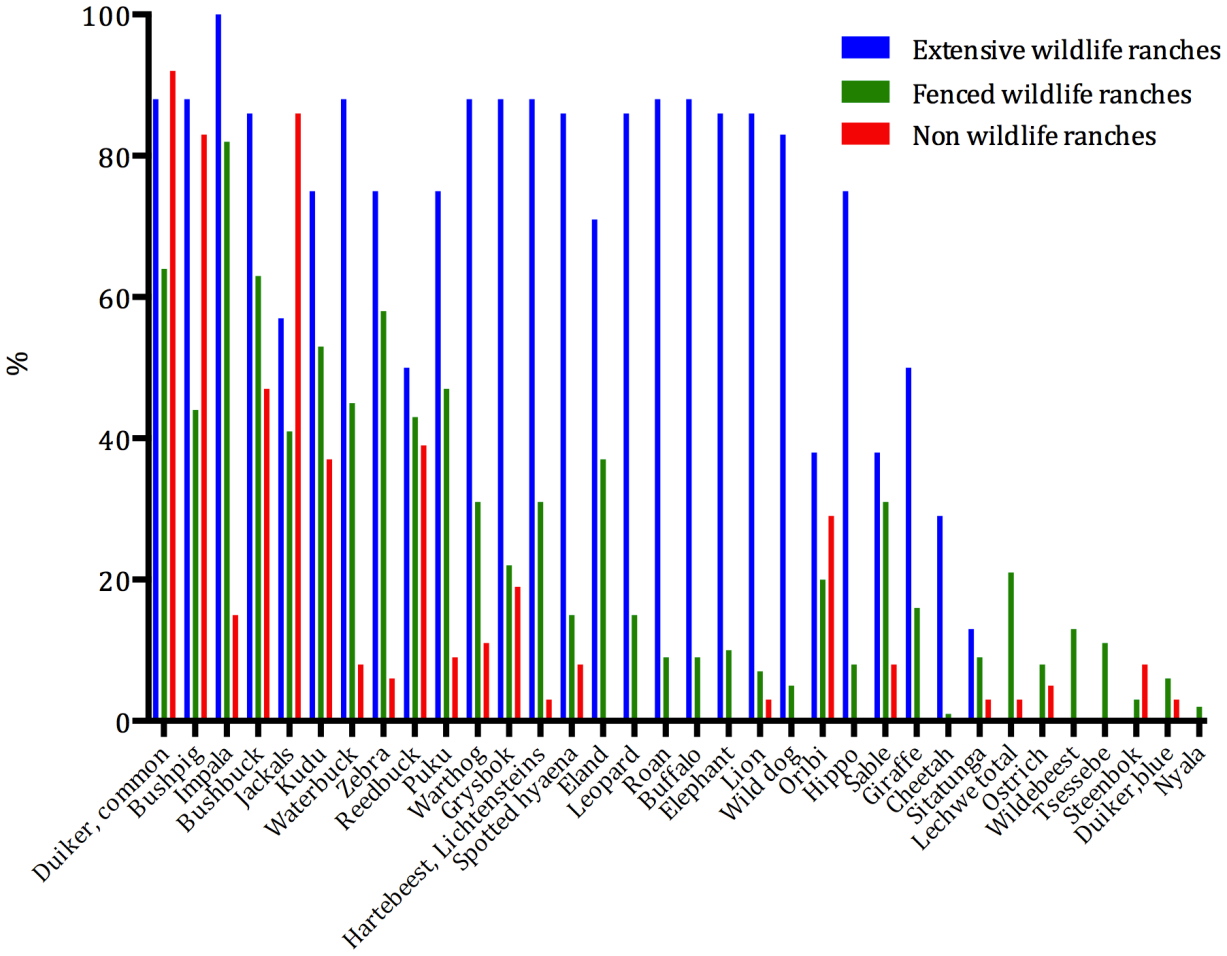
The percentage occurrence of ungulate and large predator species on Zambian ranches.

A total of 91,412 individuals of 28 ungulate species and ostriches occur on wildlife ranches (albeit including unreliable estimates of the abundance of small ungulate species or ∼63,000 excluding those species plus hippos, which are not reliably/consistently counted from the air, Table 4). The commonest species were impala *Aepyceros melampus*, kudu *Tragelaphus strepsiceros* and bushbuck *Tragelaphis scriptus* (Table 4), and the most widespread common duiker *Sylvicapra grimmia*, impala and bushbuck (Figure 2). After removing data on ungulates smaller than bushbuck, predators and hippopotamuses *Hippopotamus amphibius* (for which aerial census data were not available for protected areas), the wild ungulates on wildlife ranches comprise 16.8% of the total populations occurring in national parks, GMAs and wildlife ranches combined, despite comprising just 2.7% of the area included in the comparison (data were not available for all national parks and GMAs) (Table 4). Wildlife ranches contain significant proportions of the national populations of several species, including *inter alia* kudu (61.8%); reedbuck *Redunca arundinum* (57.9%), Eland *Taurotragus oryx* (54.4%), sitatunga *Tragelaphus spekii* (44.5%), roan *Hippotragus equinus* (29.1%), and tsessebe *Damaliscus lunatus* (27.6%) (Table 4).

**Table 4:** Estimated wildlife populations in national parks (data available for 61,462 km^2^ of the ∼64,000 km^2^), game management areas (GMAs, data available for 159,654 km^2^ of the ∼166,000 km^2^) (together ‘protected area network’) and game ranches (5,829 km^2^) in Zambia (excluding species of bushbuck size and smaller, and hippopotamuses for which count data were not available).

Species	Protected area network^a^	Game ranches^b^	% of total on gameranches	Total
Lechwe	85,545	1,513	1.7	87,058
Impala	41,327	27,998	40.4	69,325
Wildebeest	51,884	630	1.2	52,514
Buffalo	37,239	2,107	5.4	39,346
Puku	24,367	4,904	16.8	29,271
Elephant	18,924	1,710	8.3	20,634
Sable	13,067	3,682	22	16,749
Zebra, plains	9,425	2,060	17.9	11,485
Waterbuck	7,587	2,987	28.2	10,574
Kudu	3,884	6,287	61.8	10,171
Hartebeest	8,381	2,051	19.7	10,432
Roan	4,016	1,647	29.1	5,663
Reedbuck	1,989	2,735	57.9	4,724
Eland	1,306	1,558	54.4	2,864
Tsessebe	1,078	410	27.6	1,488
Giraffe	757	321	29.8	1,078
Sitatunga	409	328	44.5	737
Nyala	0	95	100	95
	311,185	63,023		374,208

a Data were obtained from: [54]–[60].

b Data obtained from ZAWA game ranch returns and questionnaire survey data.

**Figure 3:**
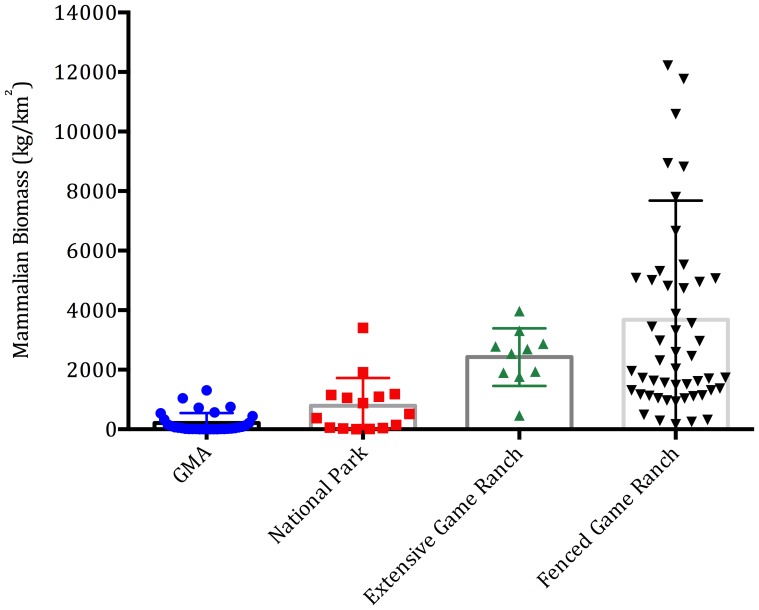
The biomass of wild ungulates (excluding species of bushbuck size and smaller and hippos, for which data were unavailable for state protected areas).

**Figure 4:**
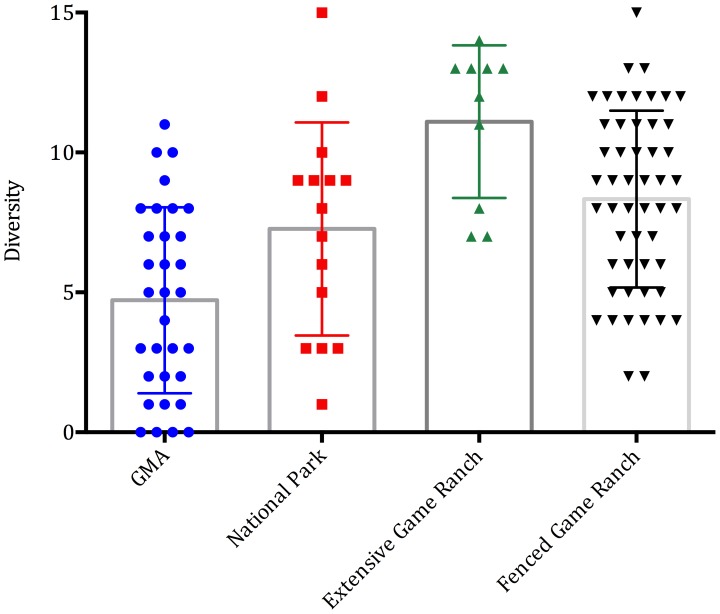
The diversity of wild ungulates (excluding species of bushbuck size and smaller and hippos, for which data were unavailable for state protected areas) include small plots as well as actual ranches).

Wildlife biomass was higher on fenced wildlife ranches (3,683±568 kg/km^2^) than extensive wildlife ranches (2,424±305 kg/km^2^), national parks (791±240 kg/km^2^) or GMAs (212±59 kg/km^2^) (*F Ratio* 11.3, d.f. = 3, p<0.001) (Figure 3). Ungulate diversity was higher on extensive wildlife ranches (11.1±0.86 species), than fenced wildlife ranches (8.34±0.45 species), national parks (7.2±0.9 species) or GMAs (4.7±0.58 species) (*F Ratio* 12.8, d.f. = 3, p<001) (Figure 4). When including all ungulates and hippos, wildlife biomass was higher on fenced (4,090±609 kg/km^2^) than unfenced wildlife ranches (3,488±759 kg/km^2^), or non-wildlife ranches (139±42 kg/km^2^) (*F Ratio* 15.8, d.f. = 2, p<0.001). The higher stocking rates on fenced game ranches are likely partly due to supplementary feed, which is provided by 50.0% of such ranches (compared to 14.2% of extensive ranchers).

The biomass of wildlife on fenced wildlife ranches comprised a mean of 52.1±42.2% of mammalian biomass, the rest being made up of livestock. The diversity of ungulates and large predators was higher on extensive wildlife ranches (20.1±1.3 species) than on fenced wildlife ranches (13.2±0.58, including small plots) or non-wildlife ranches (5.1±0.67) (*F Ratio* = 70.1, d.f. = 2, p<0.001), due to the lack of large predators and megaherbivores on the latter.

Wildlife populations were increasing on 79.2% of wildlife ranches c.f. 25.8% of non-wildlife ranches (*χ2* = 25.9, d.f. = 1, p<0.001). Increases were attributed to anti-poaching security (55.0%), good management of wildlife (15.0%). Declines were attributed to poaching (73.3%); purposeful removal of wildlife (13.3%); and human encroachment (6.6%). Whether or not wildlife populations were declining was influenced by the proportion of wildlife lost to poaching (13.4±16.3% were lost to poaching on ranches where wildlife was declining, c.f. 4.2±5.2% elsewhere) (*F Ratio* = 10.6, d.f. = 1, *p = *0.002).

### Poaching

Ninety-three percent (92.7%) of wildlife ranchers experienced poaching on their land. Thirty-four percent (33.8%) lost <10 animals per year to poaching, 21.5% lost 10–25, 16.9% lost 25–50, 10.8% lost 51–100, and the remainder >100. A mean of 8.0±17.5% of ungulate populations were lost to poaching (range 0–52.9%), the percentage being higher on non-wildlife than wildlife ranches (15.0±9.6% c.f. 5.4±8.2%) (*F Ratio* 19.0, d.f. = 1, p<0.001).

The amount of poaching was increasing on 33.3% of wildlife ranches, stable on 47.3% and declining on 19.3%. Common poaching methods included: firearms (78.2% of wildlife ranches); snares (72.7%); dogs (40.2%) and poison (15.6%). Most respondents felt that poachers hunt to obtain meat to sell (86.1%). Eighty-percent (79.3%) of wildlife ranchers employed anti-poaching wildlife scouts to protect wildlife.

Wildlife ranchers captured 2.37±0.44 (range 0–25) poachers annually and spent USD1,423±504 to process each poacher to the point of conviction (excluding anti-poaching costs), through transport and staff costs, and reward payments. Mean conviction rates were 52.6±40.6%, ranging from 0–100%. Regarding punishments allocated to poachers, 54.5% of ranchers indicated that jail terms were secured (mean length 24.4±3.8 months, range 3–72), 22.7% that fines were allocated (mean USD137±26, range USD9.9–USD395), 6.8% that a choice of jail or fine was allocated and 15.9% that poachers are never convicted. Ranchers frequently complained that poachers given prison terms were released on bail and immediately recommenced poaching, and that they typically serve a fraction of their terms. Similarly, ranchers complained that the value of fines was lower than the meat value of a single animal.

Reports of violent assault by poachers were common: poachers had shot at three ranchers and at least five wildlife scouts were reported killed by poachers in recent years. Poaching was the main reason that 54 ranchers did not renew their wildlife ranch status in 2013 (WPAZ, pers. comm.).

### Community Outreach

Fifty-seven percent of wildlife ranches were bordered by communal lands. 88.7% of these ranchers indicated that they provide benefits to communities via employment (52.9%), assistance to local schools (41.2%) and health services (15.7%) and through provision of meat (9.8%). Some have developed strong business relations with neighbouring communities. For example, one individual has developed a system whereby communities are allocated a cash dividend for each animal hunted on his property as an alternative to poaching.

### Relative Scale of the Industry

The Zambian wildlife ranching industry fares poorly relative to regional counterparts in terms of: the number of wildlife ranches; the area comprised of wildlife ranches; the amount of wildlife on wildlife ranches; the number of jobs created and the economic impact of the industry (Table 5).

**Table 5:** The scale of the regional game ranching industries (in the case of Zimbabwe, the historic scale, pre-land seizures after which many game ranches were converted into subsistence livestock farms).

	South Africa	Namibia	Zimbabwe	Botswana	Zambia	Mozambique
Total private land (km^2^)	1,006,000	356,532^a^	136,765^k^	34,904^l^	?	24,048^o^
Number of game ranches	10,000–14,000^d^	2,825^i^	1,000^k^	102^l^	129^m^	8^o^
Private land used for WBLU (km^2^)	205,000^d^	287,818^i^	27,000^k^	9,710^l^	5,829^m^	1,250^o^
% of private land used for WBLU	20.4%	80.7%^i^	19.7%	27.8%^a^	?	?
Wildlife populations on private land	18.5–20 million^e^	1.8–2.8 million^i^	841,000^k^	173,000^l^	91,000^m^	?
Economic value of the game ranching industry^a^	USD795.5m^f^	USD166m^j^	?	USD7.1m^l^	USD16.4^m^	
Value of the crocodile farming industry^g^	USD6.75m	Negligible	USD26m	Negligible	USD3-4m^n^	USD1.5m
Average number of crocodile skins exported2008–2011^h^	33,185±7,570	202±141	107,750±15,642	876±406	34,954±2,521	5,564^g^
Proportion of game ranching income derived from^b^:						
Hunting (trophy hunting, unguided hunts,meat sales)	91.8%^f^	26.1%^j^	?	54.8%	50.7%^l^	
Ecotourism	1.9^f^	66.2%^j^	?	24.9%	44.0%^l^	
Live game sales	6.3%^f^	7.7%^j^	?	20.3%	5.3%^l^	
Jobs created from game ranching	100,000^f^	?	?	1,700	2,197^l^	

a Including income from crocodile farms;

b Excluding income from crocodile farming, taxidermy and other related industries to allow for cross-country comparison.

c [69].

d [13].

e [40].

f [39].

g A Zimbabwean crocodile farmer operating in Zimbabwe, South Africa and Mozambique.

h CITES Trade Database (average values during 2008–2012, http://www.cites.org/eng/resources/trade.shtml, accessed September 2013).

i [70].

j [38].

k [6].

l Botswana Wildlife Producers’ Association, pers. comm.

m This study.

n Anonymous Zambian crocodile farmer, pers. comm. (2013).

o [71].

### Key Findings from the Financial and Economic Modelling

A small-scale fenced wildlife ranch in a mixed farming setting would yield financial rates of return of ∼15% (Table 6, Table S4). Although modest, such returns would be low risk and would diversify a farming venture. The example presented incorporated the full costs of fixed and movable assets and stock. However, some ranches are already fenced with wildlife-proof fencing and in such cases the 20-year financial rate of return would increase to 16% (Table 6). Similarly, if half the non-wildlife capital requirements were already in place, the 20-year financial rate of return would rise to 18%. If 50% or 75% of the start-up wildlife populations were already present, the projected 20-year return would increase to ∼23% and 31% respectively (Table 6). Development of an ecotourism lodge would also increase rates of return to 18% (if a lodge was built by another company on the farm and the farmer received 4% of turnover) and 25% (if the rancher built an ecotourism lodge and obtained a return of 15% of the initial investment).

**Table 6:** Results of sensitivity analysis for the financial model for small-scale fenced game ranch.

Sensitivities	Financial rates of return		
Period of analysis	10 year	20 year	40 year
**Base scenario**	**15.7%**	**15.0%**	**14.9%**
Ranch fencing already established*	17.0%	16.1%	16.0%
Half wildlife stocks already present*	25.4%	22.6%	22.2%
Three quarters wildlife stocks already present*	35.4%	31.4%	31.0%
Half non-wildlife capital already in place*	18.8%	17.8%	17.6%
Three quarters non-wildlife capital in place*	20.6%	19.4%	19.2%
Rental/royalty from a lodge (4% of lodge turnover)**	18.5%	17.8%	17.7%
Net income from a lodge (15% of lodge capital)***	25.3%	24.6%	24.6%

* Where some initial capital costs (fencing, stock capital) treated as sunk costs but still requiring maintenance and repairs and replacement.

** Where ranch leases site to an operator who pays an annual rental amounting to 4% of lodge turnover.

*** Where ranch owner develops lodge and where ranch model incorporates lodge net cash income (annual lodge net profit) as additional income only. Net cash income amounting to 15% of lodge capital investment.

Economic returns from the development of a wildlife section on a mixed ranch are estimated to be 28%, which is highly efficient (Table 6). The intensive nature of wildlife ranching on small properties ensures that the generation of value added to the national income per unit of land (at USD 12,100/km^2^ for gross national income and USD 10,800/km^2^ for net national income) is good.

## Discussion

The number and area of wildlife ranches in Zambia has increased from 30 and 1,420 km^2^ in 1997 to 177 and ∼6,000 km^2^ in 2012 [14]. The wildlife ranching industry now confers an array of benefits that fall within three categories.

### Conservation Benefits

Ungulate populations on wildlife ranches increased from 21,000 in 1997 to ∼91,000 in 2012 while those in many protected areas in Zambia and elsewhere declined steeply [20],[21],[22]. Wildlife biomass and diversity per unit area on both fenced and extensive wildlife ranches is greater than in national parks and GMAs. Wildlife ranches now protect significant proportions of the national populations of some species, and notably: reedbuck; roan; sitatunga; reedbuck; and tsessebe, which are rare and/or have restricted distributions elsewhere in southern Africa [23],[24]. We acknowledge that the aerial census data relied on for population estimates are likely to underestimate wildlife populations in protected areas to some extent. However, we have no doubt that the density of wildlife is much higher on game ranches than in protected areas on average. Wildlife ranches thus provide a reserve that can be used to re-stock protected areas, as has occurred elsewhere in the region [25],[26] and in Zambia: animals from Zambian game ranches have been used to re-stock Lusaka, Mosi-oa-Tunya, and Sioma Ngwezi national parks. This is potentially significant because the protected area estate is currently being subjected to rampant poaching [15],[16] and so many areas may require re-stocking if the threat is controlled. Wildlife ranching effectively conserves habitat in the context of rapid encroachment of portions of the protected area estate [17] and may protect habitats under-represented in the protected area network, as elsewhere in the region [27],[28]. Due to the small intensive nature of fenced wildlife ranches, and the prevalence of livestock, fenced wildlife ranches do not contribute significantly to the conservation of large predators or mega-herbivores. However, such species (including wild dogs *Lycaon pictus* and cheetahs *Acinonyx jubatus*) are effectively conserved on extensive wildlife ranches.

A benefit arising from development of the wildlife ranching industry in southern Africa has been the honing of skills associated with wildlife capture and management and the development of a generation of ecologists, wildlife veterinarians and other experts with relevant experience [9]. Such capacity is in short supply in Zambia and conservation efforts would benefit greatly from its emergence.

### Economic Benefits

Wildlife ranching and crocodile farming generate USD15.7 million for the economy, whereas the state-owned GMAs (which are ∼29 times larger) generated ∼USD16 million in 2012 [16]). Earnings per unit area and per kg of biomass from wildlife on active wildlife ranches are similar to those from livestock. Wildlife allows for utilization of land with little agricultural potential, meaning that the large areas with tsetse flies and unfertile soil could be used productively. At the individual farm level, wildlife ranching enables utilization of unfertile or rocky ground. Wildlife boosted the income of mixed-farmers by an average of 23%. A key basis for the competitiveness of wildlife-based land uses in southern Africa is the multiple income streams possible from wildlife [8]. Income from wildlife is not as reliant on rainfall and primary productivity as that from livestock and thus provides farmers with a buffer against drought [6], which is an increasingly common phenomenon in Zambia [29]. Furthermore, the diversification of income streams associated with developing wildlife-based land uses also provides buffers against changes in the prices of beef and other agricultural products. Our modelling suggests that in Zambia, as in several other southern countries in the region, attractive returns on investment from wildlife ranching are achievable [30],[31],[32].

### Social Contributions

The wildlife ranching industry provides a variety of social benefits. The industry contributes to food security through: 1) direct employment of 2,200 people; 2) investments made by ranchers in community outreach projects; 3) production of significant quantities (295,000 kg) of meat and distribution of meat to communities and workers, and 4) through foreign currency inflows from the sale of hunting and photo-graphic safaris [32]. Social benefits are perhaps highest in cases where ranchers have forged genuine business linkages with adjacent communities. Such moves also help to increase indigenous participation in the industry.

### Scope for Expansion of the Industry

There is significant scope for expansion of wildlife ranching in Zambia. Zambia is large with a relatively small and urbanized population [33]. Consequently, much of the country is sparsely populated with large areas of potentially suitable land both within and beyond gazetted GMAs. Approximately 500,000 km^2^ of forested land remains in Zambia [29], much of which is infested with tsetse fly and of limited use for livestock production. In addition, due to endemic foot-and-mouth-disease status, the export of beef and other livestock products to the European Union is not possible [34]. Several wildlife species present in Zambia are absent or rare elsewhere in the region and the biomass and diversity recorded on Zambian wildlife ranches (∼4,090 kg/km^2^ and 13 species on fenced ranches, ∼3,488 kg/km^2^ and 20 species on extensive ranches) compare favourably with those recorded elsewhere in the region (e.g. Namibian ranches 936 kg/km^2^ and 10 species) [11].

Growth in the global tourism industry has potential to stimulate expansion of wildlife ranching. Tourist arrivals to Zambia have shown strong growth in recent years [35],[36] and such trends are likely to continue, particularly given the rapid loss of wildlife in many other countries [22]. Similarly, there is significant scope for expansion of the venison industry in Zambia. In 1997, an estimated 2,500,000 kg of venison (largely sourced from poaching) was consumed in the Copperbelt and Lusaka alone, almost 10 times that currently produced on wildlife ranches [14] and there was a feeling among meat dealers that demand for venison far outstripped supply.

Wildlife ranching is more popular among young land owners, suggesting that there may be a generational shift towards the land use [11]. The number of commercial farmers has increased rapidly in Zambia during recent years and new entrants may consider wildlife to diversify and generate additional income. Finally, the climate of southern Africa is predicted to become drier and incomes from wildlife ranching will be less affected than that from livestock farming [37]. For these reasons, coupled with projected positive returns, there is a good financial case for farmers to complement livestock or crops with wildlife, particularly where they already have components of the necessary infrastructure.

### Under-performance of the Zambian Wildlife Ranch Industry

The Zambian wildlife ranching industry is growing from a small base despite many hurdles (discussed below). However, comparisons with the industries in other southern African countries provide insights into the kind of scale the industry could reach if those barriers were removed. The Zambian wildlife ranching industry compares poorly with other countries in the region in terms of the land area used for wildlife ranching, the amount of wildlife protected, jobs created and economic output [11], [38]–[40]. The Zambian wildlife ranching industry is characterized by few serious players, many wildlife ranchers not utilizing wildlife commercially, under-investment and indication that some ranchers are starting to disinvest due to frustration with policy constraints and lack of support from government. Inadequate participation by indigenous Zambians has potential to undermine the social and political sustainability of the industry if not addressed. Industry sectors prevalent elsewhere in the region, such as the breeding and sale of high-value wildlife, trade in wildlife skins, and taxidermy, are poorly developed.

### Limiting Factors and Recommended Solutions

We believe that the primary cause for the under-performance of the Zambian game ranching industry is inadequate legal protection against poaching, the lack of an adequate and appropriate legislative framework, and a system of fees and permits which create inhibitive barriers to wildlife ranching. Such barriers are largely absent for livestock production.

There is need for decisive steps and robust legislation to address poaching. Sentiments towards wildlife ranching were negative among many ranchers due to the financial costs, inconvenience and risk to personal safety arising from poaching. Bushmeat poaching in Zambia is unusual in that it is done primarily with firearms [41]. Poachers operate knowing that most game scouts are unarmed and will face legal action if they defend themselves, that the chances of capture and conviction are low and that associated penalties are weak. By contrast, penalties for stock theft are severe, a discrepancy that has been noted in other countries in the region [42]. We recommend significantly elevated punishments for poaching, equivalent to those issued for stock theft [43] or armed robbery where firearms are used, and full compensation for animals killed. Wildlife ranch status should confer the right of wildlife ranchers and their wildlife scouts to carry firearms as a means of self-defence, to arrest poachers on their property and to confiscate poaching equipment.

Amended legislation is required which outlines the parameters within which wildlife ranching may occur. That legislation should allow for a much more streamlined process for developing game ranches and provide for consolidated and reduced permitting requirements. For example, wildlife ranch status should result in the automatic allocation of safari hunting and tourism licenses. New legislation should identify and empower the Wildlife Producers’ Association as being a civic body responsible for coordinating and self-regulating the industry (B. Child pers. comm.).

A key limitation of the Zambian wildlife ranching industry is the failure to allocate complete user-rights over wildlife to landowners: ranchers are only allocated annual certificates of ownership and on unfenced wildlife ranches ownership is retained by the state. Zambia has thus not devolved user-rights over wildlife to the same degree as other countries in the region [11],[44]. In South Africa, Botswana and Namibia, complete ownership of wildlife (in Namibia just for ‘huntable species’) is allocated to a landowner if a ranch is fenced [6],[44]. In South Africa, that has been taken further, such that ownership of individually identifiable animals is retained even if escapes, is lured or is killed by a poacher (M. Boshoff, pers com.). In Zimbabwe, the 1975 Parks and Wildlife Act bestowed appropriate authority status over wildlife to land owners: however, a statutory instrument (76 of 1998) was subsequently introduced which required ranchers to apply for quotas to hunt wildlife on their land, which as discussed below was a retrogressive step designed to re-centralize control over wildlife [45].

The failure to devolve perpetual user rights over wildlife to landowners in Zambia does not provide adequate security for the significant investments needed to start wildlife ranching, and undermines the ability of wildlife ranchers to access loans. On extensive wildlife ranches, landowners are required to pay ZAWA fees for hunting wildlife (USD60–USD10,000 per animal depending on the species) and are not allowed to harvest wildlife for live sales. Investor confidence is further undermined by periodic drastic decisions that ZAWA have a tendency to make. For example, in early 2013, ZAWA imposed a hunting moratorium, which initially included extensive game ranches. Although permission to hunt was subsequently granted to extensive ranchers, uncertainty remains as to whether permission will be extended to 2014, preventing effective marketing and planning. Fenced ranchers have also been affected by the hunting ban: although they have been granted permission to hunt, they no longer benefit from the market of hunters who visit Zambia primarily to hunt in the GMAs, and who then visit ranches to collect additional species. One way to provide a more secure basis for investment for Zambian wildlife ranchers would be to introduce a clause into legislation, which identifies landowners as the indefinite ‘appropriate authority’ over wildlife that occurs on their land.

The requirement for ranchers to fence land to obtain ownership of wildlife in Zambia (and some other southern African nations) is unfortunate. Fencing is costly and frequently ecologically undesirable [43],[46]. Extensive wildlife ranches contain higher wildlife diversities and densities than national parks and GMAs and so retaining connectivity between such properties and protected areas would be beneficial for ZAWA. Furthermore, extensive ranches allow for conservation of the full range of species, including large predators. Revised legislation should allocate perpetual user rights over wildlife to all landowners with no requirement for fencing so that effective conservation by wildlife ranches is not penalised. To allay fears that extensive ranchers may then exploit wildlife coming from national parks, consumptive utilization of wildlife on such ranches could be regulated via an annual quota system approved by ZAWA.

ZAWA is a parastatal that currently relies in revenue generated from the protected area estate and from extensive game ranches to function. This model creates a conflict of interest whereby ZAWA is forced to encourage maximal utilization of resources in order to generate income for, but often to the detriment of, conservation [16]. Consequently, ZAWA has an effective monopoly over the wildlife sector, has the power to regulate its 'competitors' and it is not in their best short-term financial interests to devolve user-rights over wildlife or create an enabling environment for the private wildlife sector (B. Child pers. comm.). A similar situation arose in Zimbabwe during the late 1990s, when the wildlife authority was transformed into a parastatal required to generate their own funding a conflict of interest emerged which forced the parks authority into commercial competition with the private sector, resulting in efforts to recentralize control over wildlife resources [45]. There is need for elevated government funding for the wildlife authority in both Zimbabwe and Zambia such that such a conflict of interest does not exist and so that devolution of user-rights over wildlife to land owners does not affect their income or functionality.

Due to restrictions imposed by the National Veterinary Council, ranchers are not permitted to immobilize wildlife and so veterinarians are required for the simplest of procedures, imposing costs and delays (particularly given the acute shortage of wildlife vets in Zambia). We recommend development of a course where wildlife managers are trained to dart and immobilize wildlife safely in order to conduct simple management procedures, similar to that conducted in Zimbabwe (Zimbabwe Dangerous Drugs Act, 2001). Additionally, the controlled export of wildlife should be permitted, subject to fulfilment of veterinary protocols and wildlife regulations imposed. Live sale prices for wildlife are high [39] and prices for Zambian sub-species/races are exceptional. At present due to the ban on exports, South African wildlife ranchers have a monopoly on the sale of Zambian wildlife within that market and Zambia does not benefit at all.

Accessing land to practise wildlife ranching is difficult. Leasehold land in Zambia is scarce and costly. Ninety-four percent of Zambia is under customary ownership and the procedure for applying for title is opaque, lengthy, and requires the permission of multiple stakeholders (chiefs, local council, national government, ZAWA) [47]. There is a 5,000 ha cap on the amount of land that can be alienated [48], a size that is unlikely to be viable for extensive wildlife ranching. However, there are 167,000 km^2^ of gazetted GMAs in Zambia that are currently performing poorly due to inadequate participation of communities and uncontrolled encroachment and poaching [16]. Such areas should be made available for the development of wildlife-based land uses in the context of public-private partnerships between the private sector and communities.

Start-up costs associated with wildlife ranching are high (due primarily to the costs of erecting fencing and purchasing founder stock, in addition to construction of lodges and purchase of vehicles, etc) and likely off-putting for many ranchers, particularly given the relatively modest potential returns. However, projected economic benefits associated with wildlife ranching are high and there is thus a case for government support for the industry. Government could provide support through the provision of loans for the development of the industry and subsidization of the start-up costs. Such support should be directed particularly at increasing the participation of indigenous Zambians in the industry.

An additional constraint to development of the game ranching industry is under-performance of the ecotourism and trophy hunting industries in the country in general relative to regional competitors [49],[50]. Causes are varied but include widespread poaching of wildlife and encroachment of protected areas, high costs of air travel to Zambia, high corporate taxation, and periodic hunting moratoria, which collectively reduce visitation of the country by tourists and hunters [21],[51],[49].

Together, these constraints dissuade new entrants to the industry, discourage active participation or significant investment from current players, and encourage some ranchers to disengage. The subsequent lack of critical mass within the industry means that markets for live wildlife are small, and support services such as culling teams for harvesting wildlife for the trade in meat have not arisen, and earnings and employment are a fraction of what they could be. Addressing the constraints would significantly boost development of the wildlife ranching industry in Zambia. In addition, there is need for greatly elevated government funding for ZAWA to improve capacity to protect and manage wildlife resources [52] and a stable policy environment related to the sustainable use of wildlife.

### Relevance of Findings Elsewhere

Large-scale wildlife ranching in Africa is limited to perhaps three or four countries. There are many savanna countries with large areas of marginal land that could be potentially used for wildlife ranching, including *inter alia* Angola, Burkina Faso, Cameroon, Central African Republic, Mozambique, Sudan, and Tanzania. In many African countries protected area networks are performing poorly and wildlife ranching has significant scope for bolstering conservation efforts [22]. However, similar constraints to those observed in Zambia are likely to limit development of the industry and many of the findings of this study are likely to apply elsewhere.

## Conclusions

The Zambian wildlife ranching industry has expanded significantly in recent years and now confers substantial ecological, economic and social benefits. However, the industry is performing at a fraction of the level that it could and is being stifled by a number of key constraints. Wildlife ranching and conservation in Zambia in general would benefit greatly if these issues were addressed. A key step would be a legislative supported policy specifically developed to regulate and facilitate development of the industry. Without these changes the industry will continue to fare poorly compared to regional peers.

## Methods

This study was designed to assess the scale of the Zambian wildlife industry and to identify potentials and constraints. We did not couch the study as a comparison with livestock farming, as unlike in several other countries we do not envisage wildlife ranching replacing cattle farming where the latter occurs. Rather, we envisage wildlife ranching as having greatest potential on where livestock farming does not occur or as an accompaniment to cattle ranching.

### Literature Review

All relevant literature was reviewed after a search on Google and Google Scholar, and all relevant legislation was reviewed.

### Survey of Ranchers

A structured, pre-tested questionnaire was used in late 2012 to interview landowners to gather quantitative data on land-use, wildlife, employment and venison production on Zambian game ranches. We surveyed as many of the members of the Wildlife Producers Association of Zambia (WPAZ) and other wildlife ranchers as possible during the course of fieldwork. Ninety-seven such ranchers were surveyed whose land comprised 93.2% of the total wildlife ranching area.

For wildlife ranchers not surveyed, data on wildlife populations and wildlife utilization were obtained from Zambia Wildlife Authority (ZAWA) records. We surveyed at least 50% as many non-wildlife ranchers (i.e. livestock and/or agricultural crop farmers) as wildlife ranchers where wildlife ranches occur, to assess attitudes towards wildlife ranching. Contact details for such farmers were obtained from the Zambian Farmers Union and respondents randomly selected, yielding a sample of n = 64 non-wildlife ranchers. Lastly, stakeholders with known expertise or involvement in the wildlife ranching industry were identified and surveyed to obtain insights into the industry. These included top-ranking officials from ZAWA (*n* = 8), NGOs (*n* = 4), tourism operators (*n* = 2), the two Zambian wildlife capture teams, venison traders (*n* = 2), the sole taxidermist in Zambia, a representative from the largest tannery in the country and a representative from the Zambian Farmers Union. Refusal rate was 1.1%.

There are two broad categories of wildlife ranch in Zambia which are referred to throughout the paper: fenced ranches located primarily in the commercial farmlands between Livingstone and Mkushi, and extensive unfenced ranches alienated from customary lands elsewhere (Figure 1). Within the fenced game ‘ranch’ category there are small ‘ornamental’ game ranches (usually <2 km^2^), usually residences where wildlife is kept purely for aesthetic purposes.

### Industry Indicators

All indicators presented in this study represent the state of the industry in late 2012. Respondents were asked to estimate gross annual income from their ranch and the proportion derived from wildlife. To estimate total industry turnover, we assumed that the registered wildlife ranchers that we did not survey generated income equal to the median revenues per km^2^ reported by ranchers in the survey. We incorporated turnover estimates of the wildlife capture industry and the crocodile farming industry (from estimates provided by two major industry representatives).

We estimated meat production using data on wildlife harvest provided by landowners and for ranches not included in our survey, from ZAWA annual returns. To estimate meat production from trophy hunted animals, we used mean body masses of male animals and typical dressing percentages ([53]. For animals hunted specifically for meat, we assumed a body mass intermediate between male and female animals.

Data on workers employed for wildlife-based land uses were obtained from surveys. We applied average worker densities recorded from surveys for ranches of 0.1–1.9 km^2^; 2–10 km^2^ and >10 km^2^ to non-surveyed wildlife ranches of those sizes to estimate total employment.

Wildlife abundance was compiled from ranchers’ estimates of the number of each species on their property and from annual ZAWA returns for non-surveyed ranches. The density and diversity of wildlife in wildlife management areas and national parks were derived from [54]–[60].

### Potential Financial and Economic Output of a Model Wildlife Ranch

Ecological, financial and economic modelling was conducted to assess the potential profitability of a 20 km^2^ wildlife section developed on an existing mixed farming enterprise (following various assumptions, Table S1). To estimate carrying capacity, we took the mean large mammal biomass from 10 Zambian wildlife ranches, and multiplied the figure by 1.25 as it was unlikely that those ranches were fully-stocked. The estimated carrying capacity equated to 14.2 large stock unit equivalents per km^2^ (one large stock unit = the metabolic equivalent of a 450 kg steer [61]). We used a well-established wildlife ranch from one of the main Zambian wildlife ranching centres (Mkushi) as the basis for estimating species composition on the model ranch, using unit body masses from [62].

We assumed that 20 individuals of each ungulate species would be purchased and reintroduced, except for species where the estimated carrying capacity was <20, where the estimated maximum number that could be supported in 20 km^2^ (based on the densities observed on the five sample ranches) were assumed to be reintroduced. Additionally, we assumed that species of bushbuck *Tragelaphs scriptus* size and smaller were already present. We modelled ungulate population growth using a Ricker equation [63] where individual ungulate populations were regulated by the carrying capacity estimates for each species, and by intrinsic growth rates estimated for each species based on their mass [62]. We assumed that large predators were absent (as is typical on fenced Zambian wildlife ranches with some exceptions) and that wild ungulates would be provided with supplementary food and would thus exhibit maximal intrinsic rates of increase.

Ricker-equations depicted annual stock numbers and potential off-takes for different forms of hunting over time. We estimated annual trophy and culling quotas based on recommendations made by [64]. We derived trophy pricing from a survey of Zambian trophy operators’ websites (2012 prices, n = 10) and assumed that high-value species rare in other southern African countries (roan, sable *Hippotragus niger*, sitatunga, lechwe *Kobus leche*, hartebeest *Alcephalus buselaphus*, tsessebe) could be used to sell five-day hunts at USD1,000/day. To cater for instances where more than one such species is hunted on a single hunt, we assumed that 70% of the available trophies of each species could be used to sell hunts. We assumed that all available trophies would be hunted and that all rare or high value animals would be sold live (sable, roan, sitatunga, tsessebe) and that for the remaining species, 50% of the remaining sustainable off-take would be sold live and the remainder culled for meat. Further, we assumed that meat from trophy hunted animals would be sold at $3.6/kg and that from culled animals at $5/kg (as per market prices in Zambia).

We assessed potential financial and economic benefits associated with the model enterprise. Modelling considered only direct use-values from consumptive or non-consumptive use of wildlife. We used financial and economic enterprise models applied widely in southern Africa [65] using detailed budget/cost-benefit spreadsheets integrated with the ecological models. Annual financial income was presented for the 20^th^ year of enterprise life. Models were based on estimates of start-up costs and fixed and variable running costs derived from surveys (Tables S2, S3) to depict a typical wildlife ranching enterprise. Note that the low start up costs reported for establishing a hunting lodge may be due to the fact that hunters are generally comfortable in rustic accommodation and also because in some cases existing accommodation (sunk costs) is used or modified only slightly. Similarly in some cases estimated labour costs were low as they are shared with other land uses in many cases. We acknowledge that capital and running costs would vary greatly with conditions. To provide insights into the impacts of some such variability, we conducted sensitivity analyses to assess impacts on the return on investment of whether: the cost of reintroductions was halved or quartered (e.g. if some wildlife already existed on the property); whether an existing wildlife fence was present (as many livestock farms have existing fencing suitable for wildlife); half the requisite infrastructure was already present; and if an ecotourism lodge was developed, based on two conservative scenarios: i] if a lodge was developed by an external company and the rancher allocated 4% of turnover, and; ii] if a rancher paid for the development of a lodge and generated income equating to a 15% return on investment.

Financial cost-benefit models were run over 10, 20- and 40-year periods, and depicted annual flows of initial and replacement capital costs, variable operating costs, operating overhead costs, and gross income at constant 2012-values. Residual values for stock and depreciated capital assets were accounted for in the final year of the period analysed. Financial internal rates of return were calculated for 10, 20 and 40 years. Financial net present values were calculated for the periods at an 8% discount rate (following [66]). Financial budget models included data on initial capital costs, annual fixed and variable operating costs (including interest on capital) and annual income for the 20^th^ year of enterprise life. The budget model estimated net profit, wage bills, and production taxes such as value added tax for that year.

The model measured the contribution of the enterprise to the national economy by accounting for annual capital expenditures, economic costs of production, income, foreign inflows and outflows, and in the last year of analysis, residual values of stock, assets, and foreign debt. The budget models depicted these measures for year 20 of enterprise life. The model generated economic internal rates of return and economic net present values to reflect value added to net national income over the analysis periods, at opportunity cost.

Financial data were converted, where necessary, through shadow-pricing. Incremental national income embraces the profits and asset value gain earned in an enterprise, and all new wages and salaries, taxes net of subsidies, and returns (interest and amortization) from capital. Incremental national income is the return to the internal factors of production in the enterprise (land, labour, capital, and entrepreneurship) and the value added after expenditures on external inputs are subtracted from the gross income.

Shadow-pricing criteria developed in southern Africa [66],[67] were adapted for Zambian conditions via removal of transfers between stakeholders within the economy, inclusion of foreign payments as costs and receipt of foreign income as benefits. Wages were adjusted to reflect opportunity costs by 30% for unskilled and 60% for semi-skilled positions. A premium of 8% was allocated to tradable items to reflect excess demand for foreign exchange.

### Statistical Analyses

Questionnaire data were analysed using multiple logistic regressions, chi-squares and analyses of variance as appropriate [68]. When commencing with multiple logistic regressions or analyses of variance, all variables expected to influence the dependent variable were included in models and then removed following a backwards-stepwise procedure until all remaining variables were statistically significant. Poaching data recorded in surveys were captured as categorical responses. These data were converted to approximate proportions of the populations of ungulates on ranches by using the mid-point of each category and calculating the proportion that number comprised of the total ungulate population on each ranch. Data recorded as Zambian Kwacha are presented as USD (converted at the mean rate for 2012 of USD5,066 to 1). Means are presented ± S.D.

### Ethics Statement

The University of Pretoria Ethics Committee approved this research and approved the procedure for obtaining consent for the surveys conducted during the research. We were issued with written consent for this study from the Wildlife Producers’ Association of Zambia and the Zambia Wildlife Authority provided verbal approval and participated in the research. From respondents we obtained verbal consent prior to conducting the surveys. Written consent from individual respondents was not considered practical or necessary. We documented any cases where respondents did not wish to participate in order to calculate refusal rates.

## Supporting Information

Table S1 **Key assumptions applicable to the financial and economic small-scale fenced game ranch model (USD, 2012).**
(DOCX)

Table S2 **Schedule of typical initial capital expenditures needed at start-up on a 20 km^2^ game section, (USD 2012) (assuming that no wildlife was present at all, and a wide range of ungulate species were reintroduced).**
(DOCX)

Table S3 **Schedule of typical annual variable and fixed cost expenditures needed for the small-scale fenced game ranch model during year 20 of project life (USD 2012).**
(DOCX)

Table S4 **Results of the financial and economic model for small-scale fenced game ranch of 20 km^2^ (2012, annual figures given for year 20).**
(DOCX)
