# Supplementary material for: The Zambian Wildlife Ranching Industry: Scale, Associated Benefits, and Limitations Affecting Its Development
Source: PLoS One. 2013 Dec 18;8(12):e81761. doi: 10.1371/journal.pone.0081761 (PMC3867336; doi:10.1371/journal.pone.0081761)
Supplement: Table S1 — Key assumptions applicable to the financial and economic small-scale fenced game ranch model (USD, 2012). (DOCX) [file pone.0081761.s001.docx]

Table S1. Key assumptions applicable to the financial and economic small-scale fenced game ranch model (USD, 2012)

| **Physical Assumptions** | Unit | Quantity |
| --- | --- | --- |
| Land Extent | Km^2^ | 20 km^2^ |
| Initial Stock Purchases | Number | 302 |
| Stock on Land (by Year 20) | Large Stock Units (LSU) | 214 |
| Stock on Land (by Year 40) | Large Stock Units (LSU) | 283 |
| "Economic" Carrying Capacity | Hectares per LSU Equivalent | 7 |
| Stocking Rate (by year 20) | Hectares per LSU Equivalent | 9 |
| Stocking Rate (by year 40) | Hectares per LSU Equivalent | 7 |
| Number of Hunting Camps | Number | 1 |
| Average Management Staff | Number | 1 |
| Average Skilled Staff | Number | 1 |
| Average Unskilled Staff | Number | 3 |
| **Financial Assumptions** |  |  |
| Land Rental | USD per km^2^ | 1.26 |
| Resource Royalty | % of Sales Turnover | 0% |
| Long Term Borrowing | % of Initial Capital | 15% |
| Short Term Borrowing | % of Recurrent Costs | 20% |
| Financial discount rate | % | 8% |
| **Economic Assumptions** |  |  |
| Economic discount rate | % | 8% |
| Economic foreign exchange premium | % | 8% |
| General VAT/sales tax adjustment | % | 11% |
| Shadow wage adjustment management staff | Factor | 1.00 |
| Shadow wage adjustment skilled staff | Factor | 0.60 |
| Shadow wage adjustment unskilled staff | Factor | 0.35 |
