# Supplementary material for: The Zambian Wildlife Ranching Industry: Scale, Associated Benefits, and Limitations Affecting Its Development
Source: PLoS One. 2013 Dec 18;8(12):e81761. doi: 10.1371/journal.pone.0081761 (PMC3867336; doi:10.1371/journal.pone.0081761)
Supplement: Table S2 — Schedule of typical initial capital expenditures needed at start-up on a 20 km2 game section, (USD 2012) (assuming that no wildlife was present at all, and a wide range of ungulate species were reintroduced). (DOCX) [file pone.0081761.s002.docx]

Table S2. Schedule of typical initial capital expenditures needed at start-up on a 20km^2^ game section, (USD 2012) (assuming that no wildlife was present at all, and a wide range of ungulate species were reintroduced)

| **Capital Costs at Start-up** | **USD** | **USD/km^2^** |
| --- | --- | --- |
| **Fixed Assets** |  |  |
| Houses | 27,000 | 1,350 |
| Offices, Storerooms, Cold rooms | 32,000 | 1,600 |
| Hunting Camps Lodges | 25,000 | 1,250 |
| Boreholes, Dams, Water Reticulation | 11,650 | 583 |
| Roads, Airstrips, Firebreaks | 6,000 | 300 |
| Water Pumps | 1,500 | 75 |
| Fencing | 69,600 | 3,480 |
| Contingencies | 8,638 | 432 |
| **Movable Assets** |  |  |
| Vehicles | 55,000 | 2,750 |
| Tools, Generators, Equipment | 17,311 | 866 |
| Office Equipment | 8,976 | 449 |
| Contingencies | 8,129 | 406 |
| **Stock** |  |  |
| Initial Stock Purchases | 715,440 | 35,772 |
| Contingencies | 71,544 | 3,577 |
| **Working Capital** |  |  |
| Initial Working Capital | 16,599 | 830 |
| **Total Initial Capital** | **1,074,387** | 53,720 |
