# Supplementary material for: The Zambian Wildlife Ranching Industry: Scale, Associated Benefits, and Limitations Affecting Its Development
Source: PLoS One. 2013 Dec 18;8(12):e81761. doi: 10.1371/journal.pone.0081761 (PMC3867336; doi:10.1371/journal.pone.0081761)
Supplement: Table S3 — Schedule of typical annual variable and fixed cost expenditures needed for the small-scale fenced game ranch model during year 20 of project life (USD 2012). (DOCX) [file pone.0081761.s003.docx]

Table S3. Schedule of typical annual variable and fixed cost expenditures needed for the small-scale fenced game ranch model during year 20 of project life (USD 2012)

| **Recurrent Costs in year 20** | **USD** | **USD/km2** |
| --- | --- | --- |
| **Variable Costs** |  |  |
| Marketing – Advertising | 388 | 19.4 |
| Cost of Sales | 6,180 | 309 |
| Transport/Communications | 5,339 | 267 |
| Animal feeding | 6,601 | 330 |
| Office Supplies/Utilities | 2,022 | 101 |
| Trophy Handling/Training/Consultancies | 1,252 | 62.6 |
| Statutory Licenses | 1,138 | 56.9 |
| VAT/Sales Tax | 35,593 | 1,779 |
| **Total Variable Costs** | **58,514** | **2,926** |
| **Fixed Costs** |  |  |
| Salaries & Wages – Unskilled | 4,941 | 247 |
| Salaries & Wages – Skilled | 1,367 | 68.4 |
| Salaries & Wages – Management | 9,050 | 453 |
| Administration | 1,500 | 75.0 |
| Maintenance & Repairs | 7,179 | 359 |
| Insurance | 447 | 22.4 |
| Loan Amortization and Interest | 24,743 | 1237 |
| Provisions for Capital Replacement | 23,062 | 1153 |
| Interest on Variable Working Capital | 2,575 | 129 |
| Interest on Overhead Working Capital | 1,077 | 53.9 |
| Land Rental | 2,520 | 126 |
| **Total Fixed Costs** | **78,460** | **3,923** |
| **Total Operating Costs** | **136,974** | **6,849** |
