# Supplementary material for: The Zambian Wildlife Ranching Industry: Scale, Associated Benefits, and Limitations Affecting Its Development
Source: PLoS One. 2013 Dec 18;8(12):e81761. doi: 10.1371/journal.pone.0081761 (PMC3867336; doi:10.1371/journal.pone.0081761)
Supplement: Table S4 — Results of the financial and economic model for small-scale fenced game ranch of 20 km2 (2012, annual figures given for year 20). (DOCX) [file pone.0081761.s004.docx]

Table S4. Results of the financial and economic model for small-scale fenced game ranch of 20 km^2^ (2012, annual figures given for year 20)

| **Case** | **Small-Scale Fenced Game Ranch** | |
| --- | --- | --- |
| Value | USD or % | USD/km^2^ |
| **Financial Values** * |  |  |
| Total Initial Financial Capital | 1,074,387 | 53,719 |
| Annual Financial Turnover | 323,571 | 16,179 |
| Annual Variable Financial Costs | 58,514 | 2,926 |
| Annual Fixed Financial Costs | 78,460 | 3,923 |
| Annual Wage Bill | 15,358 | 768 |
| Annual Net Cash Income | 186,598 | 9,330 |
| Annual Resource Royalty | 0 | 0 |
| Annual Land Rental | 2,520 | 126 |
| Annual Local Community Income ** | 6,308 | 315 |
| Financial IRR (FRR @ 10 Years) | 15.7% | - |
| Financial NPV (@ 8%, @ 10 Years) | -469,181 | -23,459 |
| Financial IRR (FRR @ 20 Years) | 15.0% | - |
| Financial NPV (@ 8%, @ 20 Years) | 788,909 | 39,445 |
| Financial IRR (FRR @ 40 Years) | 14.9% | - |
| Financial NPV (@ 8%, @ 40 Years) | 1,070,231 | 53,512 |
| **Economic Values** * |  |  |
| Total Initial Economic Capital | 964,810 | 48,240 |
| Annual Economic Gross Output | 360,424 | 18,021 |
| Annual Economic Costs | 117,753 | 5,888 |
| Annual Gross Value Added (GNI) | 242,671 | 12,134 |
| Annual Net Value Added (NNI) | 216,782 | 10,839 |
| Economic IRR (ERR @ 10 Years) | 28.7% | - |
| Economic NPV (@ 8%, @ 10 Years) | 1,277,027 | 63,851 |
| Economic IRR (ERR @ 20 Years) | 27.7% | - |
| Economic NPV (@ 8%, @ 20 Years) | 1,757,448 | 87,872 |
| Economic IRR (ERR @ 40 Years) | 27.5% | - |
| Economic NPV (@ 8%, @ 40 Years) | 1,991,958 | 99,598 |
| Number of Jobs Created | 5 | - |
| Economic Capital Cost/Job | 214,402 | - |
| Domestic Resource Cost Ratio | 0.86 | - |

* IRR = Internal rate of return, FRR = Financial rate of return, NPV = Net present value, GNI = Gross national income, NNI = Net national income, ERR = Economic rate of return

** Community income assumed to be skilled and unskilled wages accruing to local residents
